# Proton transfer during DNA strand separation as a source of mutagenic guanine-cytosine tautomers

**DOI:** 10.1038/s42004-022-00760-x

**Published:** 2022-11-05

**Authors:** Louie Slocombe, Max Winokan, Jim Al-Khalili, Marco Sacchi

**Affiliations:** 1 Leverhulme Quantum Biology Doctoral Training Centre, University of Surrey, Guildford, GU2 7XH UK; 2 Department of Chemistry, University of Surrey, Guildford, GU2 7XH UK; 3 Department of Physics, University of Surrey, Guildford, GU2 7XH UK

**Keywords:** Biophysical chemistry, DNA, Computational chemistry

## Abstract

Proton transfer between the DNA bases can lead to mutagenic Guanine-Cytosine tautomers. Over the past several decades, a heated debate has emerged over the biological impact of tautomeric forms. Here, we determine that the energy required for generating tautomers radically changes during the separation of double-stranded DNA. Density Functional Theory calculations indicate that the double proton transfer in Guanine-Cytosine follows a sequential, step-like mechanism where the reaction barrier increases quasi-linearly with strand separation. These results point to increased stability of the tautomer when the DNA strands unzip as they enter the helicase, effectively trapping the tautomer population. In addition, molecular dynamics simulations indicate that the relevant strand separation time is two orders of magnitude quicker than previously thought. Our results demonstrate that the unwinding of DNA by the helicase could simultaneously slow the formation but significantly enhance the stability of tautomeric base pairs and provide a feasible pathway for spontaneous DNA mutations.

## Abstract (web summary)

Proton transfer between DNA bases can lead to mutagenic tautomers, but as their lifetimes are thought to be much shorter than DNA separation times their role during the DNA replication cycle is often overlooked. Here, the authors model the separation of the DNA base pair guanine-cytosine using density functional theory and find increased stability of the tautomer when the DNA strands unzip as they enter a helicase enzyme, effectively trapping the tautomer population.

## Introduction

In biology, the separation of a DNA duplex occurs via fraying at its terminal base pairs due to random thermal effects or the action of a helicase enzyme during the DNA replication cycle. Once the separation of DNA has started, it will likely propagate down the duplex due to the force cascading down the ribose-phosphate backbone. In the case of helicase enzymes, an active, stepping-motor action pulls on one of the strands of DNA through a narrow opening in the enzyme, thereby forcing apart the nucleobase pairs^1^.

The interaction of the non-canonical, tautomeric state of a nucleobase pair in DNA within the helicase has thus far been overlooked in the literature. Specifically, the process of DNA strand separation and its impact on proton transfer has been assumed to be simply a matter of comparing the proton transfer timescale (via quantum and classical effects) to the timescales of the biological process.

If a tautomer passes through the replication machinery, it will form a mismatch with the wrong corresponding base on the copy strand. For instance, the tautomer of guanine will pair with thymine instead of cytosine (G–C ↔ G*–C* → G*–T, where the star denotes the tautomeric non-standard form)^2,3^. Furthermore, the mismatched base pair can evade fidelity check-points of the replisome by adopting a structure similar to a Watson and Crick base pair^2,3^ resulting in an error in the genetic code and hence a point mutation.

Florian and Leszczynski^4^ first proposed that for the tautomeric mechanism to be biologically relevant, the tautomers must remain stable during the long process of DNA unwinding and strand separation, which are the prerequisite steps for the synthesis of the new DNA strand by the polymerase. Consequently, the lifetimes of the tautomers should exceed this characteristic time for the base-pair opening ( ~ 10^−10^*s*)^4^.

In the last decade, numerous authors^3,5–10^ argued that the tautomers’ lifetime is much shorter than the helicase separation time. Therefore, no tautomeric population would successfully survive the DNA strand separation by the enzyme. If the G–C tautomer has a short lifetime and reverts to the standard canonical form, the potentially mutagenic point defect is rendered ineffective during the uncoiling process. Subsequently, the tautomer is not propagated into the two single-stranded DNAs. On the contrary, if the tautomeric lifetime is longer than the double-strand separation time, the tautomeric form will survive the biological process. Under closer inspection, the timescale reasoning requires further justification and refinement. Here, we will unpick some of the core assumptions and provide evidence for the need for a more careful investigation of enzyme effects on the DNA tautomers.

In the following sections, we first use quantum chemical models to determine the effect of an induced separation of the two strands of DNA on the structures of the G–C and G*–C* dimers and on the characteristics of the minimum energy pathway linking the two endpoints between the bases. We find that the features of the proton transfer are quasi-linearly correlated with the separation distance. To accompany our quantum chemistry calculations of the G–C dimer, we also evaluate the occurrence of separation events in classically simulated aqueous DNA subjected to a small separation force. We find a wide variety of opening events but reveal a characteristic separation speed unaffected by choice of steering force.

## Results

**Fig. 1:**
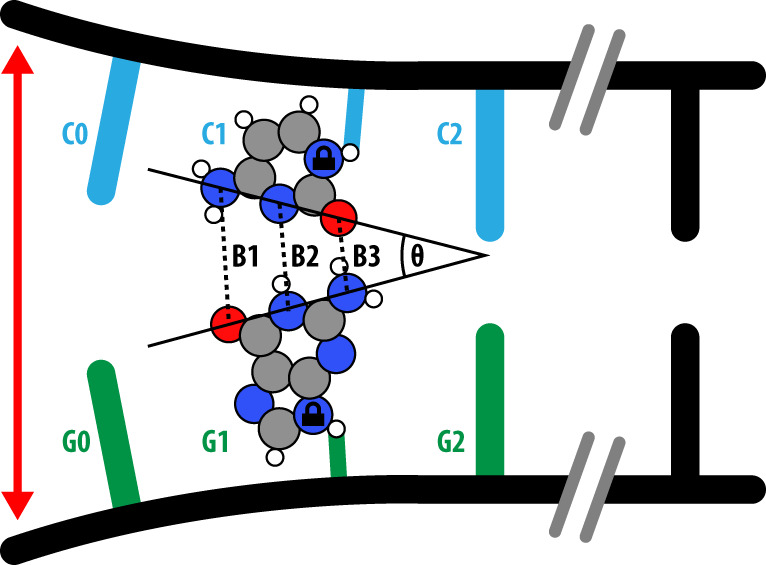
The separation scheme used to investigate how the canonical and tautomeric G–C base-pairs separate. Four G–C base pairs of the 14 base-pair DNA duplex used in the molecular dynamics simulations are shown, with a separating force (red arrow) applied to the first base pair’s (G0–C0) backbone. DFT calculations were performed only on base-pair G1–C1, where atoms marked with the lock icon were fixed. We define the three hydrogen bonds; Bond 1 (B1) as the distance measured from DG:O^6^-DC:N^4^, Bond 2 (B2) from DG:N^1^-DC:N^1^, and Bond 3 (B3) DG:N^2^-DC:O^2^. The opening angle *θ* measures the asymmetry with which the hydrogen bonds stretch.

We model the separation of the DNA bases using density functional theory (DFT) at the B3LYP+XDM/6-311++G**^11^ level of theory (NWChem^12^) with an implicit solvent. In the DFT calculations, we truncate the model to the G–C dimer, constrain the R-group atom (where the base would join the rest of the DNA), and separate the bases. Figure 1 provides a summary of the scheme. See the Methods section for further information on the separation methods.

**Fig. 2:**
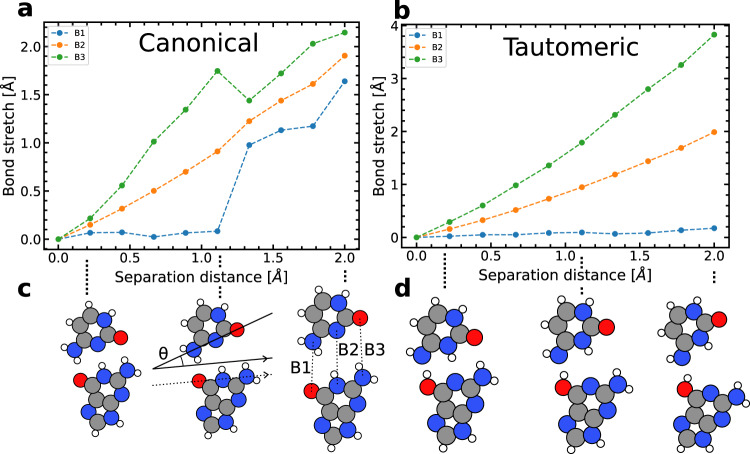
Bond length dependency on the separation distance of the G–C dimer. Here, the separation distance is defined as the distance between the non-hydrogen bonded atoms participating in the hydrogen bonds) calculated by DFT. **a** The stretching of the canonical form of G–C from their unconstrained equilibrium lengths. The equilibrium lengths for the canonical base are 2.89 Å, 2.96 Å, 2.89 Å for B1, B2, and B3, respectively. **b** The stretching of the bonds of the tautomeric form of G–C, where two hydrogens have transferred. The equilibrium lengths are 2.61 Å, 2.89 Å, 3.01 Å for the tautomeric base. Whereas **c** and **d** show the structural changes of the G–C base’s canonical and tautomeric forms, respectively. During separation, the bond lengths and angle significantly change.

We systematically vary the separation distance between the bases and study the effect of this splitting on the hydrogen bond lengths and energies. Figure 2 shows the structural changes of the G–C base /as a result of the induced separation distance. For the canonical form Fig. 2a, c, initially, there are no visible changes to the structure other than the elongation of the hydrogen bonds holding the bases together. However, as the separation distance increases, the two bases undergo an internal rotation relative to each other (measured by the angle *θ* in Fig. 1). The rotation helps to minimise the length of one of the O–H–N hydrogen bonds (B1 or B3) while the other two bonds are stretched. In the DFT calculations, there is a clear preference for the O–H–N hydrogen bond (B1) to maintain its equilibrium length while the base rotates. The non-uniformity of the separation implies that the bases do not synchronously split apart, but instead separate asymmetrically. The rotation about the fixed R-group is physically consistent since it is the only covalently bonded link between the base and the rest of the DNA.

A non-linear change in the bonding angle suggests that the proton transfer mechanism is fundamentally different from the idealised equilibrium picture previously modelled^3–10^ (see references within), where the bases are assumed to be largely unaffected by the pulling of the helicase enzyme. However, these calculations indicate that the helicase–DNA interaction cannot be ignored and requires further investigation.

A comparison between the canonical vs. the tautomeric form is shown in Fig. 2a, b, demonstrating that there is some significant difference between the rotation of the base, depending on where two of the three hydrogen bond protons are located. Here, the O–H bond of the tautomeric G offers a much more comprehensive hydrogen bonding range due to being on the outer edge of the molecule—in comparison to the standard form of C. As a result, the O–H bond of the tautomeric G remains in a hydrogen bond for much longer as the separation distance increases.

The bond length stretching is further highlighted in the bottom panels of Fig. 2. The panels show the change in the length of each hydrogen bond in the canonical G–C and tautomeric form. For the canonical case, the O–H–N bond is shown to stay relatively constant during the separation until 1.2 Å. After this point, it begins to stretch in line with the other hydrogen bonds. Whereas for the tautomeric form, the top bond is essentially not involved in the breaking until a separation distance of 2.0 Å. Further details can be found in Supplementary Note 1.

### Dynamics of the separation process

Building upon our DFT calculations, we explore the separation dynamics of a G–C base pair within a more extensive model system comprising aqueous double-stranded DNA, with 14 base pairs in total. For these calculations, we apply a steering force during a Molecular Dynamics (MD) simulation to model the external action of a helicase enzyme. Computational details are available in the Methods section. The pulling force was applied between the backbone atoms of the first G–C base pair to increase the likelihood of separation during the simulations. The three hydrogen bond lengths (B1, B2, and B3 in Fig. 1) of the base pair in question were analysed over a range of MD replicas to gather statistics on the separation dynamics. A large number of distinct but short lived fluctuations are observed, mimicking the breathing of DNA. Should these fluctuations possess properties independent of the steering force, we can argue that they are characteristic of DNA strand separation, and thus also transferable to enzymatic action.

**Fig. 3:**
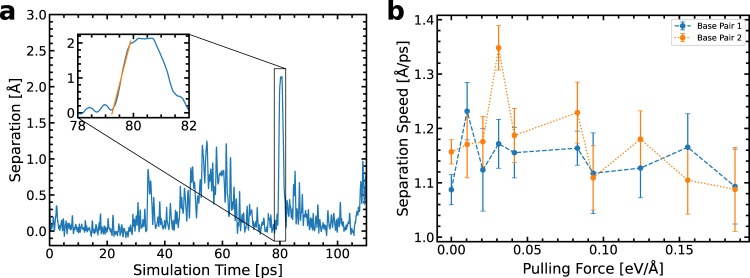
The procedure for estimating the base-pair separation speed. **a** Demonstrating a separation event during a 200 ps molecular dynamics simulation on the time series of the base-pair separation (blue solid line). The inset figure includes a linear line of best fit (orange solid line) of the separation event to determine the speed. **b** The arithmetic mean and standard error of separation speeds for a range of forces taken from a sample of 210 molecular dynamics simulations containing *n* = 1442 separation events for base pair G1–C1 (blue error bars joined by blue dashed line) and for base pair G2-C2 (orange error bars joined by orange dashed line).

Figure 3 provides an example fit of a separation time series, as well as the resulting statistics across all our dynamics simulations. While separation speed varies in a complicated manner with the pulling force, there is a significant overlap of the standard error of adjacent base pairs and forces. Thus, we conclude that the separation dynamics occur with a separation speed of approximately 1.2 Å ps^−1^, without a significant correlation to the force or base pairs. Now satisfied that we are not introducing significant bias, we turn our attention to the atomistic mechanism of the separation events.

Following the previous timescale hypothesis^4^, and assuming that, after 2.0 Å of separation, no reverse proton transfer occurs (see Supplementary Note 2), the tautomer’s lifetime must exceed ~ 1.7 ps. This requirement is two orders of magnitude shorter than the quoted characteristic time for the base-pair opening ~ 100 ps^4^. Descriptions of the mechanism of DNA strand separation by helicase enzymes are informed by the rate at which the enzyme translocates DNA by measuring the number of base pairs processed by the enzyme in a short period^1,13–17^. This ignores the possibility that individual stages of the helicase’s dynamics occur considerably faster. Our atomistic view of the separation shows that splitting individual base pairs is much quicker than the overall speed of the helicase action since it does not include translocation activity. While the tautomer might not outlive a complete cycle of helicase’s stepping-motor action, we propose that for it to be mutagenic it needs only to outlive the time taken to separate the base pair up to 2.0 Å. Our results demonstrate that the separation of a base pair due to external action occurs within a timescale relevant to that of the proton transfer. Further on in this letter, we explore the implications by considering the effect of base-pair separation on the energetics of the tautomerisation via double proton transfer and reconsider the relevancy of the timescale.

### Opening angles

**Fig. 4:**
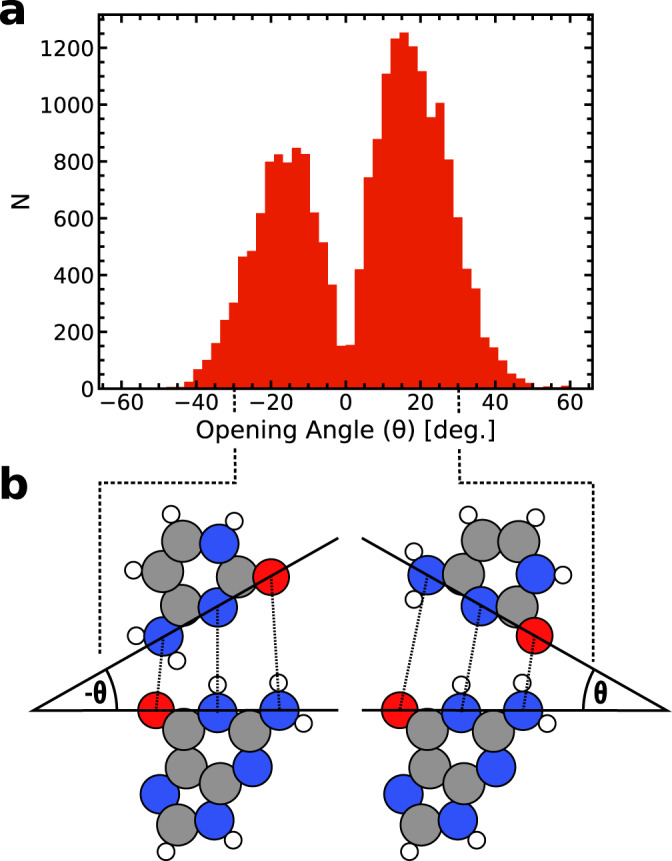
The statistical distribution of opening angles. **a** The statistical distribution of opening angles during steered molecular dynamics in base pairs 1 and 2 (red filled histogram bars). Negative angles suggest that B1 stays fixed while B3 opens. The static analysis with DFT suggests an opening angle of –22 degrees is energetically favourable without thermal effects. **b** Example of two snapshot geometries from MD runs, highlighting the direction of the opening angle (*θ*).

To further clarify the complicated way in which an external force separates DNA, an opening angle (*θ*) was defined for each separation event (see Fig. 1 and Methods section). Figure 4 demonstrates a bimodal distribution, peaked at *θ* = (18.9 ± 0.1)^∘^ and (−17.4 ± 0.1)^∘^, with only a few events showing synchronous, symmetric stretching of the three hydrogen bonds (zero opening angle). Consequently, the separation dynamics does not occur in a perfectly symmetric fashion as generally assumed^4,5^ and instead, an asymmetric breaking mechanism between the two DNA strands is much more probable (97.8% of events have ∣*θ*∣ > 3^∘^), with a clear preference for specific opening angles, which have the minimum energy requirements. In comparison to our DFT calculations, the introduction of the backbone fluctuations and thermal ensemble induces a bias towards one or the other of the opening directions. 39% of the trajectories follow a path that closely follows the direction described by the DFT result, where the system is restrained at the R-group. For the negative opening angle distribution (the one consistent with the DFT picture), and given the structure of the G–C base pair, an opening angle magnitude of (−17.4 ± 0.1)^∘^ suggests that the length B1 stays fixed while B3 is stretched by approximately ~ (1.4 ± 0.4) Å. This value compares well to the DFT geometry optimisations (see Fig. 2), which predicts B3 to stretch up to ~1.7 Å (relative to B1) before B1 is dilated. This suggests that the bond angles of the DFT calculations on the single base pairs are consistent with the MD picture, which includes the larger structure. However, it is unclear how the larger structure influences the proton transfer.

The bimodal distribution in the DNA separation events demonstrates a diverse and rich environment of energetic scenarios that are radically different from the idealised assumption made by previous authors, who either reduce the problem to a comparison of lifetimes disregarding the mechanisms of strand separation^4,7,9,10,18,19^ or perform their calculations only in the static aqueous dimer^4,5,18,20–22^. Although several authors have pointed to the fact that the complex external environments may strongly determine the influence of tautomers on mutation^3,6,8,23^, our MD results show just how diverse the biological environment experienced by DNA is.

### Proton transfer

**Fig. 5:**
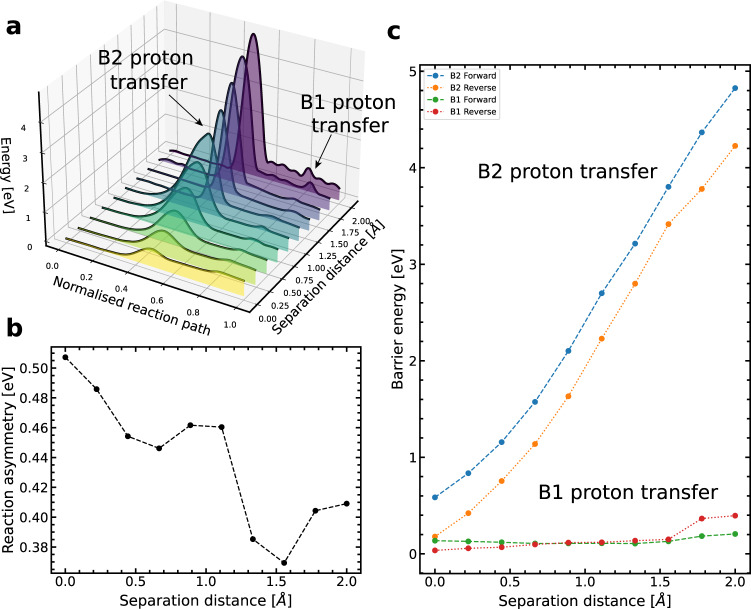
The double proton transfer tautomerisation reaction pathway. **a** Double proton transfer tautomerisation as a function of separation distance and the reaction path image. **b** Demonstrating the changes in the reaction asymmetry as a function of the base separation distance. **c** The changes in the forward and reverse reaction barriers of the canonical to tautomeric double proton transfer scheme in G–C as a function of the imposed separation distance. The plotted barrier energy is the transition state energy referenced to either the canonical, tautomeric or intermediate (single proton transfer) stable state.

For each separation distance, we perform an analysis of the double proton transfer scheme using a machine learning approach to the nudged elastic band algorithm^24,25^, which yields the minimum energy path and determines the transition state of the reaction. We connect the canonical to the tautomeric form producing an energy landscape for the double proton transfer, see Fig. 5a and Supplementary Note 1 and 2.

We define the reaction energy asymmetry as the energy difference between the canonical G–C and the double proton transfer, tautomeric G*–C* product. In Fig. 5b, the asymmetry is displayed as a function of the separation reaction coordinate. Initially, the reaction asymmetry corresponds to the unconstrained calculation (0.51 eV) at the equilibrium distance. As the separation distance increases, the asymmetry drops to a minimum of 0.37 eV at 1.5 Å. From 0 to 1.0 Å the reaction asymmetry briefly dips and then rises; this is due to a complex interplay between the local rearrangement of atoms, the bonding configuration, and the rotation of the base about the aforementioned R-groups. At separation distances greater than 1.5 Å, the asymmetry begins to increase.

The proton transfer pathway can be described as follows. Initially, we observe the O–H bonds stretch with little to no rotation or buckling of the overall structure of either base; instead, the bases remain essentially facing each other (*θ* = 0. 0^∘^). We note that the B2(N–H–N) proton moves first, followed by the B1(O–H–N) proton. However, as the separation distance increases, both bases rotate, in the opposite sense, during the reaction pathway to minimise the bond lengths (see figures in Supplementary Note 1). The general reaction pathway changes so that the bases rotate after the first proton transfer, preceded by another transfer. For more information, see Supplementary Note 1.

We observe a two-step transfer process where the middle hydrogen initially moves (B2), followed by the B1 hydrogen. Thus, the reaction path comprises two energy barriers, indicating the presence of a stable single proton transfer intermediate between two transition states. The intermediate corresponds to a structure in which only the B2 hydrogen atom has moved from G to C. Conversely, there is no stable intermediate corresponding to a single proton transfer for the B1 hydrogen bond to move. Figure 5c summarises how the two barriers change with separation distance. The B2 hydrogen reaction barrier is very sensitive to the separation distance, and it rapidly grows with increasing separation (from 0.57 eV to 4.80 eV). On the other hand, the B1 hydrogen reaction barrier stays approximately constant for distances below 0.9 Å due to the bases rotating, keeping the top bond at the equilibrium length. While the B1 hydrogen reaction barrier is constant during the initial separation, then as the base distance increases, it increases rapidly as the bond lengthens.

Gheorghiu et al^9^. have observed both the concerted and stepwise proton transfer mechanism, while others, including Brovarets et al.^7^ and Slocombe et al.^10^, only observe a concerted mechanism. Gheorghiu et al.^26^ found that the proton transfer mechanisms varied during the ensemble quantum mechanics/molecular mechanics simulations and that the concerted DPT mechanism for G–C is only a small subsample of a more extensive collection of viable mechanisms: double proton transfer, concerted vs. stepwise, single proton transfer, concerted vs. rearrangement. Instead, for G–C, the stepwise process dominates with a probability of 0.84 vs. 0.12 for the concerted mechanism due to the interaction with the larger DNA structure and local solvent environment. Gheorghiu reports that the first reaction barrier is (0.61 ± 0.05) eV and the second (0.07 ± 0.03) eV and a reaction asymmetry of (0.59 ± 0.05) eV.

In this study, we found that at the global minimum (no separation distance), there is a reaction asymmetry of 0.507 eV, which is slightly larger than in our previous work^10^ due to the interactions with the solvent and the incorporation of dispersion corrections. The first barrier has energy 0.574 eV, and the second barrier has energy 0.516 eV. Thus, there is a 0.058 eV reverse barrier from the double to single proton transfer product. The single proton transfer minimum has an energy of 0.399 eV relative to the canonical form.

Consequently, during the cleavage process, the energetic landscape of the reaction will change as a function of time. As a result, the reaction barrier and the energy difference between the reactant and products would drastically change. The change in the energetic landscape could also depend on the timescale of the separation rate compared to the period of the vibrations in G–C. Provided that the vibrational modes of the bases are similar or quicker than the timescale of the separation, the bases will have time to rearrange during the separation as calculated here. The rearrangement during the separation must then be incorporated into the model determining the rate. Conversely, if the separation is quicker than the system’s dynamics, one can assume that all the atoms are stuck in place while the bases dissociate (frozen approximation).

On the other hand, Slocombe et al.^23^ demonstrated there is a continuous exchange of the canonical and tautomeric forms due to the fast reaction rates, in turn, due to a significant quantum component. While the lifetime of a single tautomer might be short, the formation rate (forward reaction rate) might be high, such that over an ensemble of bases passing through the helicase, a proportion of them pass. During the separation process, the canonical reactant is continuously forming the product. Consequently, it is a combined process that competes with the separation timescale.

Including a more comprehensive description of the cellular environment (stacking and solvent effects) has recently been suggested to alter the reaction asymmetry^6^ such that the tautomeric state vanishes on the free energy surface. Such interactions could avert the tautomer’s formation and prevent it from potentially leading to a mutation. Gheorghiu et al^9^. suggest coupling to the larger environment offers a diverse ensemble of reaction pathways. To investigate this, we took a snapshot from an MD run presented above and re-determined the reaction path when including the rung below the separating base (pair G2-C2 in Fig. 1). In the calculation we assume that the base above is fully separated and thus, we can omit it from our simulation system as it no longer introduces stacking interactions with the base in question. Furthermore, we assume a separation of timescales whereby the base, which begins to separate would relax, while the base below would remain largely unchanged by the base separation. This assumption is further justified in Supplementary Note 3. Using the same methods as described before, at 0.39 Å separation distance the first energy barrier for proton transfer has an energy of 1.08 eV. This can be compared to 1.06 eV at the same distance but with two isolated bases. The second barrier has an energy of 0.098 eV (vs. 0.120 eV with the isolated base pair), with a reaction asymmetry of 0.467 eV (vs. 0.462 eV with the isolated base pair). The second reaction barrier shows the largest difference to the PES of the single base-pair proton transfer, with a change of 0.02 eV. This finding is consistent with Das et al.^27^, who conducted MD simulations and showed that adjacent base-pair stacking modifies the proton transfer profile on the order of 0.04 eV.

**Fig. 6:**
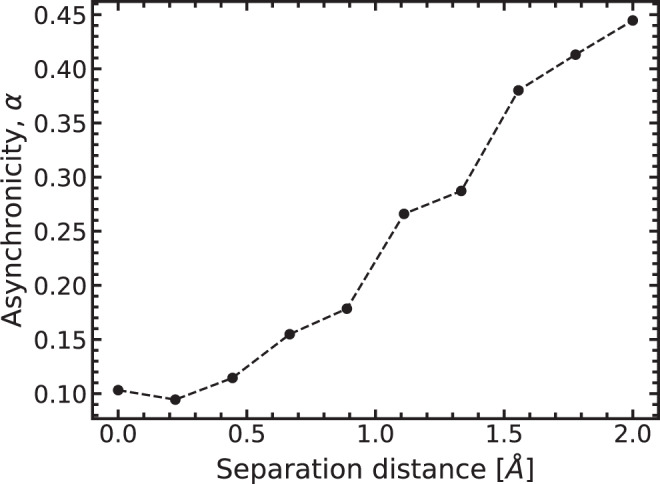
Measuring the asynchronicity as the DNA bases disassociate. Here, the asynchronicity (*α*, black circles joined by black dotted line) is calculated for each double proton reaction and displayed as a function of induced separation distance.

Figure 6 displays the asynchronicity as a function of the separation distance induced between the DNA bases. Asynchronicity is a measure of the separation between the two proton transfer events; further detail can be found in the methods section and Supplementary Note 4. Here, we use asynchronicity to quantify how the proton transfer mechanism changes during the base dissociation process. Figure 6 demonstrates that the double proton transfer initially has some concurrence indicated by a low asynchronicity value. However, as the bases are further apart, the first and second proton transfer becomes an increasingly separate event. The disconnection of the two proton transfer events along the reaction path could lead to a distribution of outcomes of product states. Furthermore, the increased asynchronicity indicates an increased localisation of the single proton transfer. Consequently, the single proton transfer could also occur along with the double proton pathway. However, due to the prohibitively significant initial forward reaction barrier, the population of either product becomes increasingly unlikely as the DNA base is further separated by the replication machinery.

On the other hand, proton-coupled electron and hole transfer (PCET) is a prominent feature of radiation-induced excited-state dynamics and subsequent DNA damage^28^. The current theoretical framework of PCET has successfully treated these types of problems^29,30^. For example, applying PCET to the excited-state dynamics of the one-electron oxidation of G–C decouples the proton and electron transfer from the middle hydrogen bond N of G (B2 in our labelling) to the N on C^28^. Similarly, a PCET excited-state deactivation mechanism for G–C has been proposed from experiment and theory (see review Kumar et al.^28^). Furthermore, Femtosecond transient absorption spectroscopy suggests that the excited-state PCET between the DNA stands has a pronounced deuterium isotope effect^31^. These findings match the transition state calculations presented in this paper, as we determine the same B2 proton transfer pathway.

## Discussion

In this work, we analysed the double proton transfer rate during DNA strand separation and proved that a simple comparison of the tautomeric lifetime is insufficient to determine the survival probability of the tautomer during this process. We propose that the proton transfer potential is not static; instead, the synchronicity of the transfer process and the activation barriers for each proton transfer drastically change as the DNA strands are pulled apart. For the G–C base, we observe both rotation and internal rearrangement of the bond lengths to minimise the energy requirement during the breaking of the hydrogen bonds as the bases split; this has a profound effect on the proton transfer energy landscape. As a result, the double proton transfer mechanism becomes an asynchronous and stepwise process with two different and well-defined reaction barriers. In particular, we observe a quasi-linear dependence of the energy of the first barrier on the separation distance. Consequently, the G*–C* tautomeric state becomes more stable as the hydrogen bonds break. The overall reverse barrier of the proton transfer (G*–C* → G–C) increases rapidly as a function of the separation distance between G and C; this yields a drastic increase in the lifetime of the potentially mutagenic tautomer (G*–C*). On the other hand, the forward barrier (G–C → G*–C*) also increases as a function of separation distance. Thus, although the survival lifetime of G*–C* increases dramatically during the process of DNA strand separation, the overall probability of trapping a G*–C* tautomer is probably extremely low. At the equilibrium distance, there is debate over a metastable G*–C* state^9^, while there is a substantial energy barrier for larger separation distances.

Consequently, we determine that a direct comparison between the biological timescale and the lifetime of the tautomeric state is misleading when making assertions about tautomeric populations becoming mutations. Furthermore, we suggest that the method of determining the proton transfer rate kinetics needs to be revised since the system is out of equilibrium. Instead, a well-parameterised time-dependent kinetic model is required to describe the low initial population and its subsequent trapping.

In summary, the work here only scratches the surface of describing the biology involved in producing the mutations from the proton transfer mechanism. However, we have gone further than the status quo and laid the path forward to accurately determining the mutation mechanism. Finally, to fully answer whether G–C tautomers lead to the point mutation during DNA replication, we underscore the requirement to combine a time-dependent kinetic model to resolve the competing biological splitting timescale with calculations of the interaction between helicase and the tautomer.

## Methods

### Modelling the separation process using density functional theory

We model the separation process using DFT methods. We use NWChem 7.0.2^12^ at the B3LYP+XDM/6-311++G** level of theory. We use the B3LYP exchange-correlation functional^11^ with exchange-hole dipole moment (XDM), a non-empirical dispersion scheme^32,33^ to account for long-scale dispersion relations we expect to play a more dominant role as the bases are further apart. We pick XDM over other models since it offers greater accuracy and flexibility at reasonable computing cost^34^. Recently, Gheorghiu et al.^26^ have benchmarked the optimum combined exchange-correlation functional, basis, and dispersion correction and determined that the combination provides fair agreement with higher levels of theory at a reasonable computational expense.

For the DFT calculations, we embed the DNA bases in an implicit continuum solvation model^35–37^ with a low dielectric factor. We use a dielectric factor of *ϵ* = 8.0^38,39^, describing the combined influence of the surrounding water molecules and protein interface, which we expect to see when the DNA interacts with the helicase.

We performed an unconstrained geometry optimisation of the canonical and tautomeric forms of G–C using the L-BFGS algorithm^40^ implemented in the atomic simulation environment (ASE)^41,42^. All the structures were optimised using a force tolerance of 0.01 eV Å^−1^.

We define the separation reaction coordinate as the distance between the constrained R-groups of the bases (where the base would join onto the rest of the DNA). With this reaction coordinate defined, we can move the bases apart by shifting the bases some distance along the separation coordinate. In reality, the separation reaction coordinate is likely not a straight line due to the interactions with the DNA backbone and enzyme, restricting the movement of the bases. We first limit our system size and focus on getting the QM calculation accurate as a first approximation. Molecular dynamics investigations of DNA duplex separation provide insight into the random nature of these fluctuations and their timescales.

If we allow enough time for the bases to relax during the separation process, we can optimise the geometry of the base at each separation distance. During the optimisation, we apply a constraint to the R-group where the base joins the backbone. The geometry is allowed to relax at each separation distance while the coordinate of the R-group atoms is fixed. The constraints prevent the system from drifting back together and simulate the strain imposed on the base from the rest of the DNA separation. The rationale for fixing the R-group is that the separation forces originate from the backbone and propagate to the base via the R-group. We can determine the reaction asymmetry by repeating the calculation for the canonical and tautomeric states.

### Obtaining the reaction pathway

We obtained the potential energy landscapes describing the proton transfer reactions using a machine learning approach to the classical all-nudged elastic band algorithm (ML-NEB)^24,25^. The ML-NEB approach minimises the number of DFT single-point energy calculations required to accurately depict the minimum energy path. In our treatment, we collect the movement of the protons transferring (and other atoms moving to facilitate the transfer) into a single axis. The reaction pathway contains a general description of the transfer process; the energetic landscape of this pathway is then explored using ML-NEB. The ML-NEB algorithm incorporates a Gaussian regression model to produce a surrogate description of the accurate minimum energy path. Thus, the uncertainty in the energy points on surrogate minimum energy path becomes the convergence criteria.

ASE^41,42^ was used throughout this work to connect NWChem to Python3 and the ML-NEB algorithm. All pathway calculations are optimised to a force tolerance of 0.01 eV Å^−1^, with a maximum uncertainty on each image to be 0.02 eV. To increase the resolution of the reaction path while keeping the computational time down, we perform the ML-NEB calculations in two steps. After relaxing the pathway using the 15 images, we interpolate between every image and insert a new image, bringing the total number of images to 29. We then relax the extended pathway, providing a higher resolution of the reaction path.

### Molecular dynamics

Molecular dynamics were performed in GROMACS 2018^43^. The 14 base-pair B-DNA duplex system was constructed from two identical chains of single-stranded DNA with the sequence: C^3^CCACGTACGTGGG^5^. Surrounded in a box of explicit SPCE solvent extending 2 nm in each cartesian direction, and sodium ions to neutralise the system. The force field used for the DNA was CHARMM36^44^. Several replica systems were minimised, equilibrated, and simulated with a pulling force acting on the backbones of the first base pair. For each replica the system was first minimised to a maximum force of 12 kj mol^−1^ nm^−1^, the equilibration took place over 500 ps of NVT ensemble with 1 fs timestep, and a temperature of 310 K maintained via a Nose-Hoover thermostat with coupling constant of 0.2 ps. In excess of 50 ns of simulation data was collected and analysed, distributed across 66 replicas with 10 different forces.

To gather statistics on the separation dynamics, the three hydrogen bond length time series of the base pair in question were analysed. The bond length time series were initially passed through a Savitsky-Golay filter with window size 63 and polynomial order 2. A separation metric was defined as the arithmetic mean of the hydrogen bond extensions relative to their equilibrium value. This separation metric was studied across each MD run, in the many instances where the separation peaked above the noise floor, a least squares regression was performed with a linear function whose slope is used to estimate the separation speed of the separation event in question. The fit was limited to the first two Angstroms of separation, as beyond this the hydrogen bonds are deemed to be broken. The uncertainty in the slope of the linear function provides a metric for the quality of the fit. Linear best fits with a negative slope, and those with relative uncertainty above 5 percent were discarded.

To classify the asymmetry behaviour of each separation event, opening angle was defined from the dot product between the vectors connecting the donor/acceptor atoms of each nucleobase, i.e., for guanine: ${{{{{{{\bf{G}}}}}}}}=\vec{{{{\mbox{DG:N}}}}^{2}{{{\mbox{DG:O}}}}^{6}}$, and for cytosine: ${{{{{{{\bf{C}}}}}}}}=\vec{{{{\mbox{DC:O}}}}^{2}{{{\mbox{DC:N}}}}^{4}}$. To distinguish between opening with the top bond opening first and bottom bond staying fixed, and vice versa the cross product of the two vectors was calculated and a negative value was applied if it was anti-aligned with the direction of the double helix.

### Proton transfer asynchronicity

To further analyse how the proton transfer mechanism changes during the base dissociation process we determine the asynchronicity (*α*) of the double proton transfer. As a concept, asynchronicity is defined by a slight separation of the two proton transfers, i.e., one proton transfers, other heavy ions rearrange and then the second proton transfers. We formally define asynchronicity as

1 $$\alpha =\frac{\left|{\alpha }_{{{{{{{{\rm{B1}}}}}}}}}-{\alpha }_{{{{{{{{\rm{B2}}}}}}}}}\right|}{\left|\left|{q}_{{{{{{{{\rm{IRC}}}}}}}}}\right|\right|}.$$

where,

2 $${\alpha }_{i}={{{{{{{\rm{argmax}}}}}}}}\left(\frac{\partial {x}_{i}}{\partial {q}_{{{{{{{{\rm{IRC}}}}}}}}}}\cdot \frac{\partial {x}_{i}}{\partial {q}_{{{{{{{{\rm{IRC}}}}}}}}}}\right).$$

Here, *x*_*i*_ is the Cartesian vector of atom *i* and *q*_IRC_ is the reaction coordinate. The partial derivative of the Cartesian vector tracks the motion of, say, B1 or B2 along the reaction coordinate. The dot product normalises the B1 or B2 motion relative to the collective rearrangement of all atoms. If there is no motion of atom *i*, it does not contribute to the reaction path, then *α*_*i*_ → 0. If *α*_B1_ ~ *α*_B2_ the protons transfer at the same point on the reaction coordinate, thus the process is synchronous. While when *α* > 0, one proton moves before another, and larger values of *α* indicate a large separation of the transfer events. In the extreme case when the protons transfer at each opposing end of the reaction coordinate, *α* tends to unity.

## Supplementary information

Winokan_PR File

Supplementary Information

## Data Availability

The data presented in the figures of this article are available from the corresponding authors upon reasonable request. The reaction pathways and structures are available on Github.

## Code availability

The analysis source codes are available on Github.

## References

[1] Yu J, Ha T, Schulten K (2006). Structure-based model of the stepping motor of PCRA helicase. Biophysical J..

[2] Watson JD, Crick FHC (1953). The structure of DNA. Cold Spring Harb. Symp. Quant. Biol..

[3] Kim Y (2021). Quantum biology: an update and perspective. Quantum Rep..

[4] Florián J, Leszczyński J (1996). Spontaneous DNA mutations induced by proton transfer in the guanine ⋅ cytosine base pairs: an energetic perspective. J. Am. Chem. Soc..

[5] Jacquemin D, Zúñiga J, Requena A, Céron-Carrasco JP (2014). Assessing the importance of proton transfer reactions in DNA. Acc. Chem. Res..

[6] Soler-Polo D, Mendieta-Moreno JI, Trabada DG, Mendieta J, Ortega J (2019). Proton transfer in guanine-cytosine base pairs in b-DNA. J. Chem. Theory Comput..

[7] Brovarets’ OO, Hovorun DM (2019). Atomistic mechanisms of the double proton transfer in the h-bonded nucleobase pairs: Qm/qtaim computational lessons. J. Biomolecular Struct. Dyn..

[8] Srivastava, R. The role of proton transfer on mutations. *Front. Chem*. **7**, https://www.frontiersin.org/article/10.3389/fchem.2019.00536 (2019). 10.3389/fchem.2019.00536 PMC6712085 31497591

[9] Gheorghiu A, Coveney P, Arabi A (2020). The influence of base pair tautomerism on single point mutations in aqueous dna. Interface focus.

[10] Slocombe L, Al-Khalili JS, Sacchi M (2021). Quantum and classical effects in DNA point mutations: Watson-crick tautomerism in at and gc base pairs. Phys. Chem. Chem. Phys..

[11] Becke AD (1993). Density-functional thermochemistry. iii. the role of exact exchange. J. Chem. Phys..

[12] Apra E (2020). Nwchem: past, present, and future. J. Chem. Phys..

[13] Lohman TM, Bjornson KP (1996). Mechanisms of helicase-catalyzed dna unwinding. Annu. Rev. Biochem..

[14] Dillingham MS, Wigley DB, Webb MR (2000). Demonstration of unidirectional single-stranded dna translocation by pcra helicase: measurement of step size and translocation speed. Biochemistry.

[15] Cox K, Watson T, Soultanas P, Hirst JD (2003). Molecular dynamics simulations of a helicase. Proteins: Struct. Funct. Bioinforma..

[16] Toseland CP, Martinez-Senac MM, Slatter AF, Webb MR (2009). The ATPase cycle of pcra helicase and its coupling to translocation on DNA. J. Mol. Biol..

[17] Park J (2010). Pcra helicase dismantles reca filaments by reeling in dna in uniform steps. Cell.

[18] Villani G (2006). Theoretical investigation of hydrogen transfer mechanism in the guanine–cytosine base pair. Chem. Phys..

[19] Brovarets’ OO, Hovorun DM (2014). Why the tautomerization of the g ⋅ c watson-crick base pair via the dpt does not cause point mutations during dna replication? qm and qtaim comprehensive analysis. J. Biomolecular Struct. Dyn..

[20] Gorb L, Podolyan Y, Dziekonski P, Sokalski WA, Leszczynski J (2004). Double-proton transfer in adenine- thymine and guanine- cytosine base pairs. a post-hartree- fock ab initio study. J. Am. Chem. Soc..

[21] Cerón-Carrasco J (2009). Intermolecular proton transfer in microhydrated guanine- cytosine base pairs: A new mechanism for spontaneous mutation in DNA. J. Phys. Chem. A.

[22] Pérez A, Tuckerman ME, Hjalmarson HP, Von Lilienfeld OA (2010). Enol tautomers of watson-crick base pair models are metastable because of nuclear quantum effects. J. Am. Chem. Soc..

[23] Slocombe L, Sacchi M, Al-Khalili J (2022). An open quantum systems approach to proton tunnelling in DNA. Commun. Phys..

[24] Hansen, M. H. et al. An atomistic machine learning package for surface science and catalysis. Preprint at 10.48550/arXiv.1904.00904 (2019).

[25] Torres JAG, Jennings PC, Hansen MH (2019). Low-scaling algorithm for nudged elastic band calculations using a surrogate machine learning model. Phys. Rev. Lett..

[26] Gheorghiu, A. Ensemble-based multiscale modelling of DNA base pair tautomerism in the absence and presence of external electric fields. Ph.D. thesis, UCL (University College London, 2021).

[27] Das S, Nam K, Major DT (2018). Rapid convergence of energy and free energy profiles with quantum mechanical size in quantum mechanical–molecular mechanical simulations of proton transfer in DNA. J. Chem. Theory Comput..

[28] Kumar A, Sevilla MD (2010). Proton-coupled electron transfer in DNA on formation of radiation-produced ion radicals. Chem. Rev..

[29] Weinberg DR (2012). Proton-coupled electron transfer. Chem. Rev..

[30] Tyburski R, Liu T, Glover SD, Hammarström L (2021). Proton-coupled electron transfer guidelines, fair and square. J. Am. Chem. Soc..

[31] de La Harpe K, Crespo-Hernández CE, Kohler B (2009). Deuterium isotope effect on excited-state dynamics in an alternating gc oligonucleotide. J. Am. Chem. Soc..

[32] Johnson ER, Becke AD (2006). Van der waals interactions from the exchange hole dipole moment: application to bio-organic benchmark systems. Chem. Phys. Lett..

[33] Becke AD, Arabi AA, Kannemann FO (2010). Nonempirical density-functional theory for van der waals interactions. Can. J. Chem..

[34] Otero-De-La-Roza A, Johnson ER (2013). Non-covalent interactions and thermochemistry using xdm-corrected hybrid and range-separated hybrid density functionals. J. Chem. Phys..

[35] Klamt, A. & Schüürmann, G. Cosmo: a new approach to dielectric screening in solvents with explicit expressions for the screening energy and its gradient. *J. Chem. Soc*. Perkin Trans. 2799–805. 10.1039/P29930000799 (1993).

[36] York DM, Karplus M (1999). A smooth solvation potential based on the conductor-like screening model. J. Phys. Chem. A.

[37] Marenich AV, Cramer CJ, Truhlar DG (2009). Universal solvation model based on solute electron density and on a continuum model of the solvent defined by the bulk dielectric constant and atomic surface tensions. J. Phys. Chem. B.

[38] Pitera JW, Falta M, van Gunsteren WF (2001). Dielectric properties of proteins from simulation: the effects of solvent, ligands, ph, and temperature. Biophysical J..

[39] Li L, Li C, Zhang Z, Alexov E (2013). On the dielectric "constant” of proteins: smooth dielectric function for macromolecular modeling and its implementation in delphi. J. Chem. Theory Comput..

[40] Payne MC, Teter MP, Allan DC, Arias TA, Joannopoulos JD (1992). Iterative minimization techniques for ab initio total-energy calculations - molecular-dynamics and conjugate gradients. Rev. Mod. Phys..

[41] Larsen AH (2017). The atomic simulation environment–a python library for working with atoms. J. Phys.: Condens. Matter.

[42] Bahn SR, Jacobsen KW (2002). An object-oriented scripting interface to a legacy electronic structure code. Comput. Sci. Eng..

[43] Berendsen H, van der Spoel D, van Drunen R (1995). Gromacs: a message-passing parallel molecular dynamics implementation. Comput. Phys. Commun..

[44] Hart K (2012). Optimization of the charmm additive force field for dna: Improved treatment of the bi/bii conformational equilibrium. J. Chem. Theory Comput..

