# Supplementary material for: Proton transfer during DNA strand separation as a source of mutagenic guanine-cytosine tautomers
Source: Commun Chem. 2022 Nov 5;5:144. doi: 10.1038/s42004-022-00760-x (PMC9814255; doi:10.1038/s42004-022-00760-x)
Supplement: Supplementary file 2 — Supplementary Information [file 42004_2022_760_MOESM2_ESM.pdf]

Supporting Information: Proton Transfer During DNA Strand Separation as a Source of Mutagenic Guanine-Cytosine Tautomers

Louie Slocombe,[1,2,*] Max Winokan,[1,†] Jim Al-Khalili,[3,‡] and Marco Sacchi[2,§]

[1]*Leverhulme Quantum Biology Doctoral Training Centre, University of Surrey, Guildford, GU2 7XH, UK.*
[2]*Department of Chemistry, University of Surrey, Guildford, GU2 7XH, UK.*
[3]*Department of Physics, University of Surrey, Guildford, GU2 7XH, UK.*
(Dated: October 5, 2022)

This material contains supplemental information for the results presented in the manuscript *Proton Transfer During DNA Strand Separation as a Source of Mutagenic Guanine-Cytosine Tautomers.* The data presented in the figures of the article are available from the corresponding authors upon reasonable request. The reaction pathways and structures are available on Github. Furthermore, the analysis source codes are also available on Github.

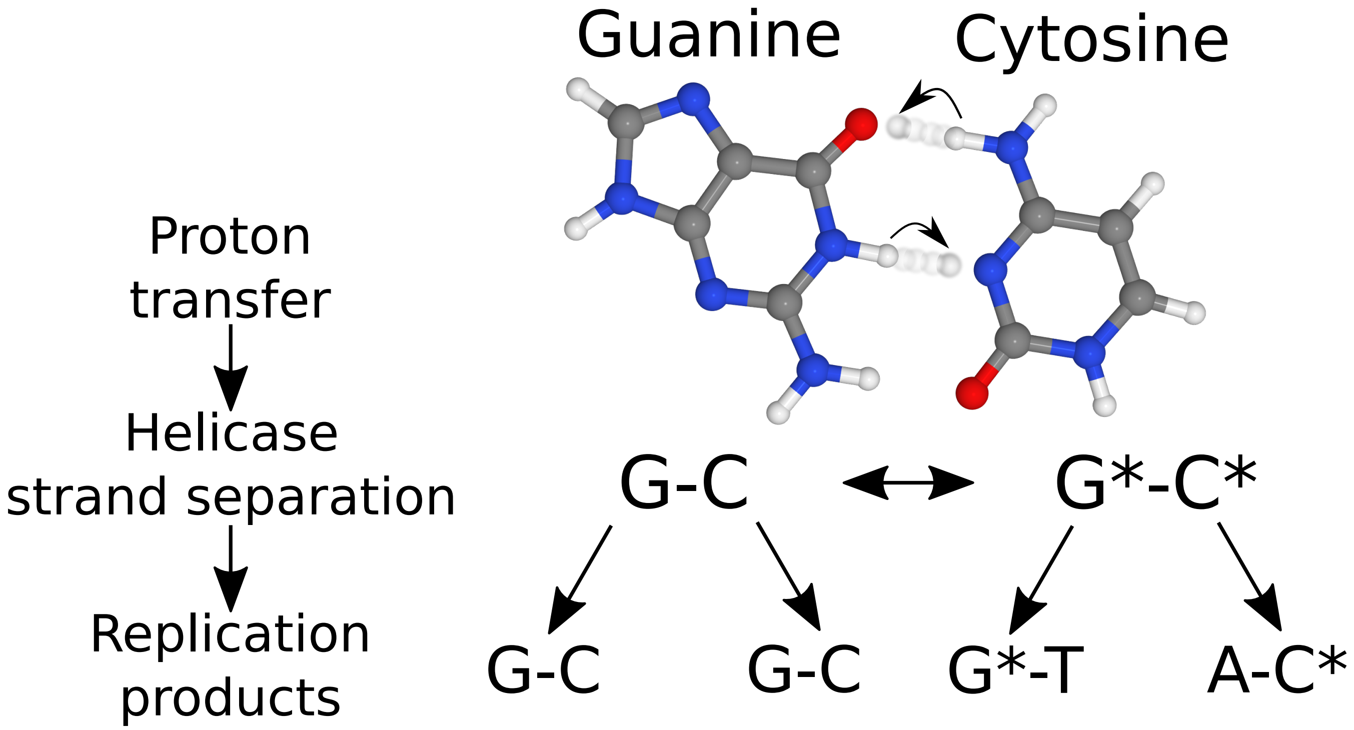

Supplementary Figure 1: The double proton transfer reaction along the hydrogen bonds between the G-C base pair. The scheme depicts the resulting products of this transfer. If the tautomers pass through the strand separation their daughter strands mismatch with the wrong base pair.

* louie.slocombe@surrey.ac.uk
† m.winokan@surrey.ac.uk
‡ J.Al-Khalili@surrey.ac.uk
§ m.sacchi@surrey.ac.uk

CONTENTS

Supplementary Note 1: Further Reaction Pathway Analysis 2

Supplementary Note 2: Proton Transfer at Larger Separation Distances 4

Supplementary Note 3: Justifying Assumptions Made in Stacking Calculations 6

Supplementary Note 4: Further Notes on Proton Transfer Asynchronicity 7

SUPPLEMENTARY NOTE 1: FURTHER REACTION PATHWAY ANALYSIS

Reading from left to right, each plot in a row of Fig. 2 shows the image number of the reaction path. We use 15 images along the reaction pathway, the first corresponding to the canonical form and the last to the tautomeric form. In each row, the seventh image is the transition state. Each row corresponds to a reaction path for a given separation distance. Thus reading down, we observe how the proton transfer landscape changes as the bases separate.

Fig. 2 shows the splitting of the canonical and tautomeric form of G-C while being constrained at the R-group mentioned before. Initially, there are visible changes other than the elongation of the hydrogen bonds holding the bases together. As the separation distance increases, there is a slight internal rotation of the bases relative to each other. The rotation minimises the length of the hydrogen bonds. There is a clear preference for the top hydrogen bond to maintain its equilibrium length while the base rotates. The rotation only happens since we are fixing the R-group, where the base joins onto the sugar and the rest of the DNA backbone. The R-group is where the base will be pulled from since this is the only covalently bonded link between the base and the rest of the DNA.

In comparison, there is some difference between the rotation of the base for the canonical vs the tautomeric form (see the bottom panel of Fig. 2). Here, the O-H bond of the tautomeric G offers a much more comprehensive hydrogen bonding range due to being on the outer edge of the molecule - in comparison to the standard form of C. As a result, the O-H bond of the tautomeric G remains in a hydrogen bond for much longer as the separation distance increases.

In reality, the splitting reaction coordinate is likely not a straight line due to the interactions with the DNA backbone and local water environment, restricting the movement of the bases. Consequently, we expect too see a range of opening angles as observed by molecular dynamics calculations.

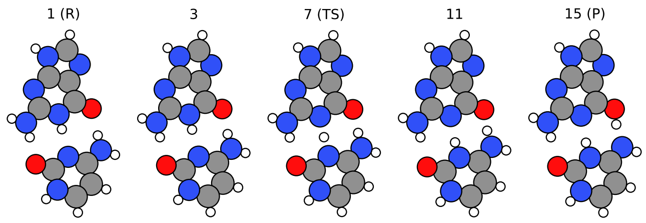

(a) 0.0 Å

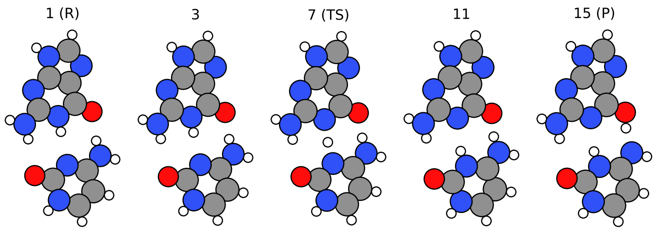

(b) 0.444 Å

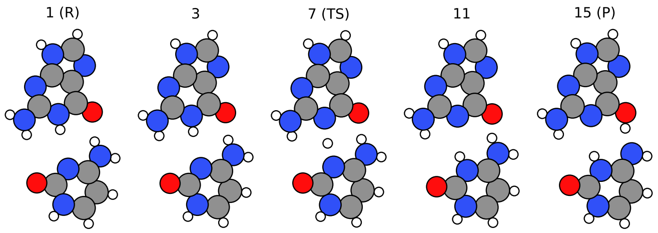

(c) 0.889 Å

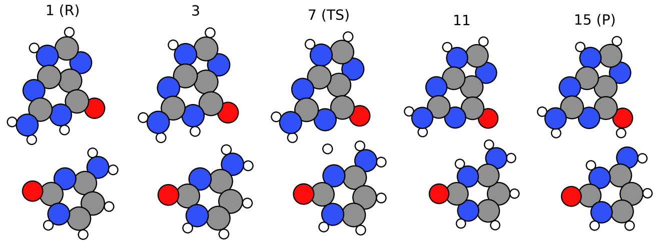

(d) 1.33 Å

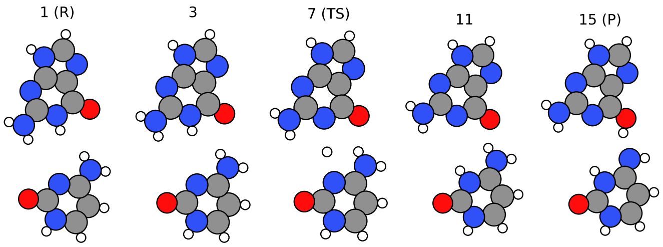

(e) 1.78 Å

Supplementary Figure 2: The double proton transfer reaction pathway. Each row is the reaction path, and each column is the pathway over increasing separation distances. The R label corresponds to the reactant, TS, the transition state, and P the product.

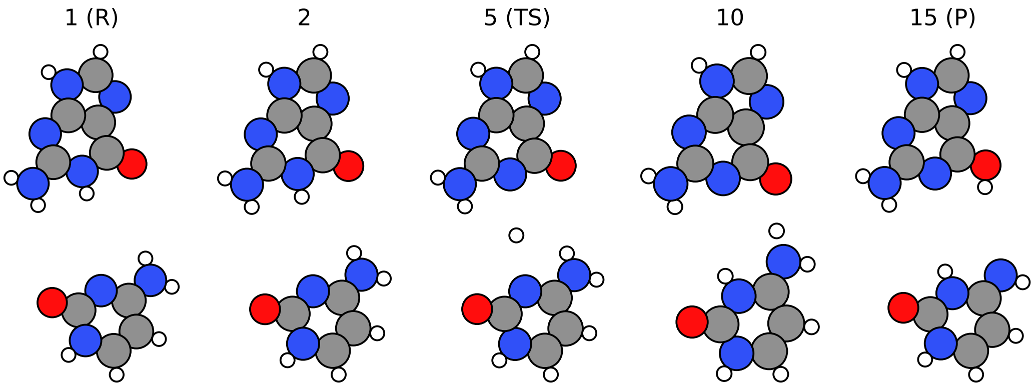

(a) 2.5 Å

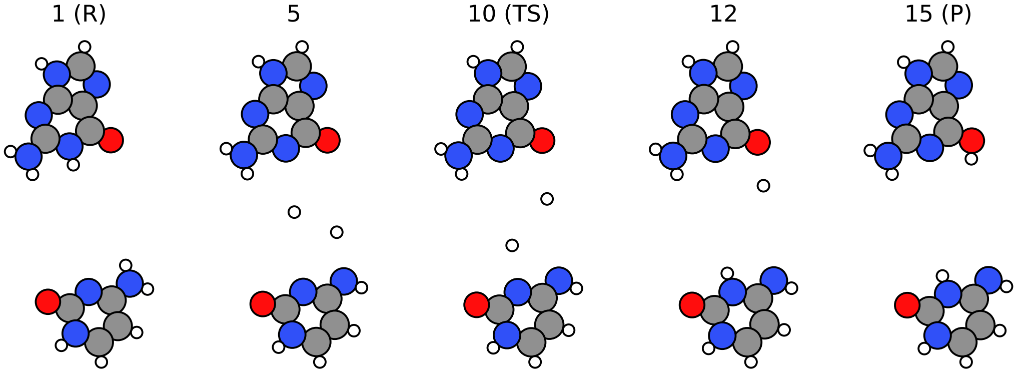

(b) 5.0 Å

Supplementary Figure 3: The reaction pathway at large separation distances. Each row is the proton transfer reaction pathway, and while each column shows the mechanism at increasing separation distances.

SUPPLEMENTARY NOTE 2: PROTON TRANSFER AT LARGER SEPARATION DISTANCES

We explore the double proton transfer mechanism at separation distances greater than > 2.0 Å.

Fig. 3 highlights the reaction pathway of two separation distances, 2.5 Å and 5.0 Å. In both pathways, the rotation is diminished as it no longer becomes favourable to rotate to minimise bonding length, but to maintain the tail end of the hydrogen bonds so that the residual bonds are maximised. In the 2.5 Å case, the pathway is comprised of two separate movements of the protons. First, the middle hydrogen transfers, followed by the top proton in a similar mechanism observed before. On the other hand, for 5.0 Å the middle hydrogen begins to transfer before meeting the other halfway between the bases. This switch to a concerted asynchronous mechanism is likely due to the solvent's increasing role when the bases are far apart, since at this distance a water molecule could begin to creep in between the bases.

In Fig. 4 the energy of the reaction paths are shown. At a separation of 2.5 Å, two distinct large barriers are observed. The first barrier corresponds to the middle hydrogen moving. The single proton transfer has a deep well due to the high barriers on the reaction coordinate in both directions. In addition, it has a considerable asymmetry value of 0.668 eV relative to the canonical form. The second barrier corresponds to the top proton transferring. At this separation distance, it is the minimum energy path, but not the most energy efficient mechanism. Instead, we expect the intra-base transfer scheme, which is not considered in the reaction path calculation, to compete with this mechanism since it has a much lower and narrower reaction barrier. While at a separation of 5.0 Å the reaction barrier has doubled in height compared with that of the 2.5 Å separation, and the barriers have merged. We expect both the classical and quantum contribution to the rate to be low, resulting in a slow reaction that is unlikely to form biological products in either of these cases. Consequently, we expect proton transfer to be unlikely to occur above 2.0 Å.

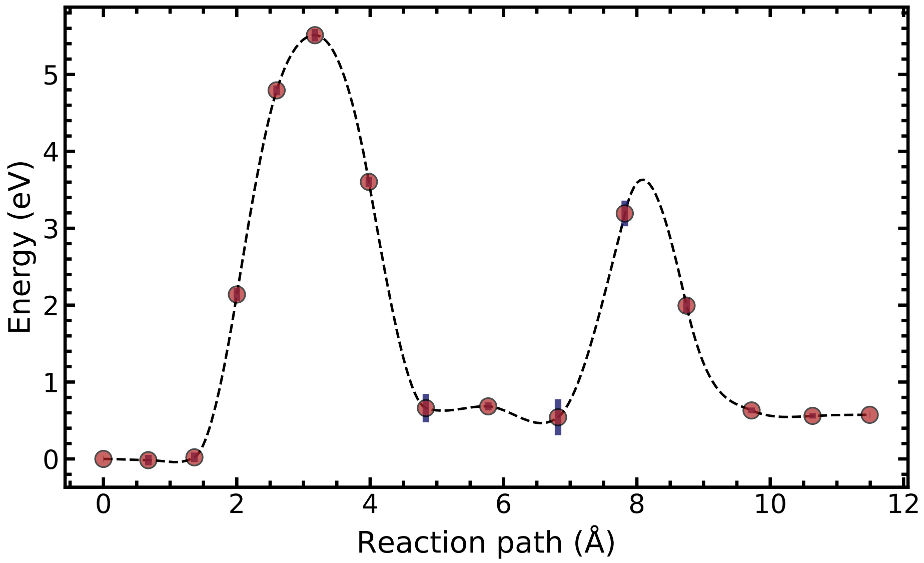

(a) 2.5 Å

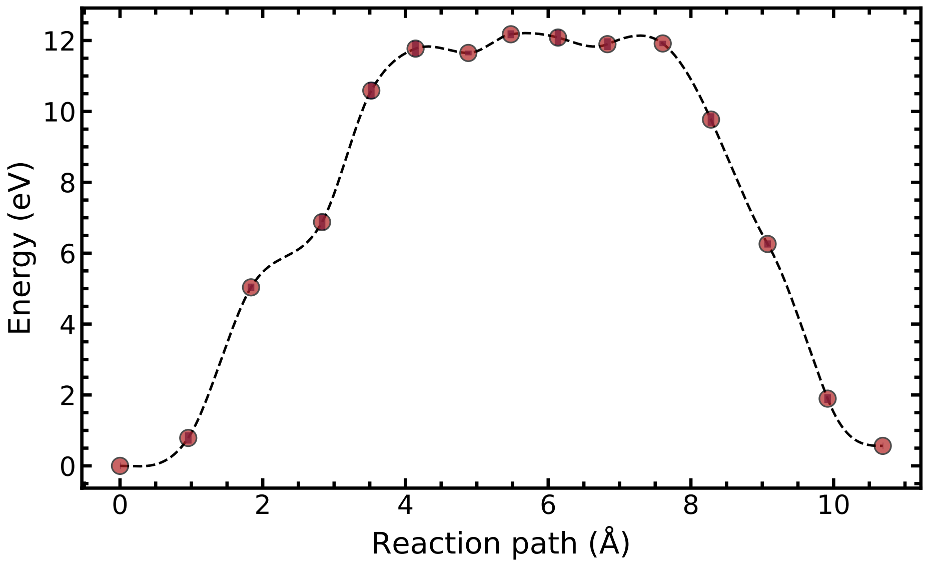

(b) 5.0 Å

Supplementary Figure 4: Reaction paths at separation distances larger than 2.0 Å.

SUPPLEMENTARY NOTE 3: JUSTIFYING ASSUMPTIONS MADE IN STACKING CALCULATIONS

During our quantum mechanical calculations where the effect of stacking interactions between DNA base pairs on the double proton transfer was investigated, the base pair below the base pair of interest was assumed to be frozen during the separation event in the base pair above it. The resulting reaction profile is shown in Fig. 5. This assumption was not taken lightly, and backed up by our molecular dynamics simulations, in which we can see a clear separation of timescales between the opening of one base pair, and the next. This can be deduced from Fig. 6 where the separation of base pair 2 (plotted on the y-axis) remains near-zero during separation events in base pair 1 (x-axis).

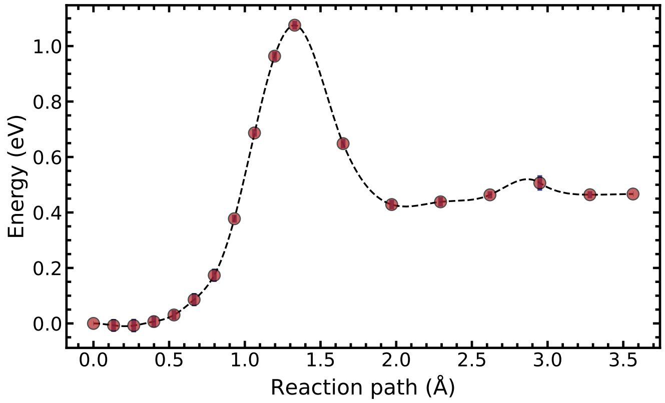

Supplementary Figure 5: The double proton transfer reaction pathway including the stacking interactions from a frozen base pair below. The separation distance is fixed to0.39 Å.

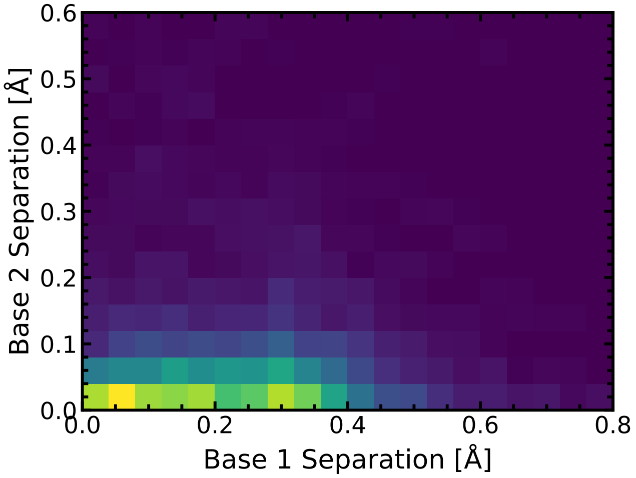

Supplementary Figure 6: The separation of base pair 2 during a separation event in base pair 1 in our Molecular Dynamics simulations of aqueous DNA.

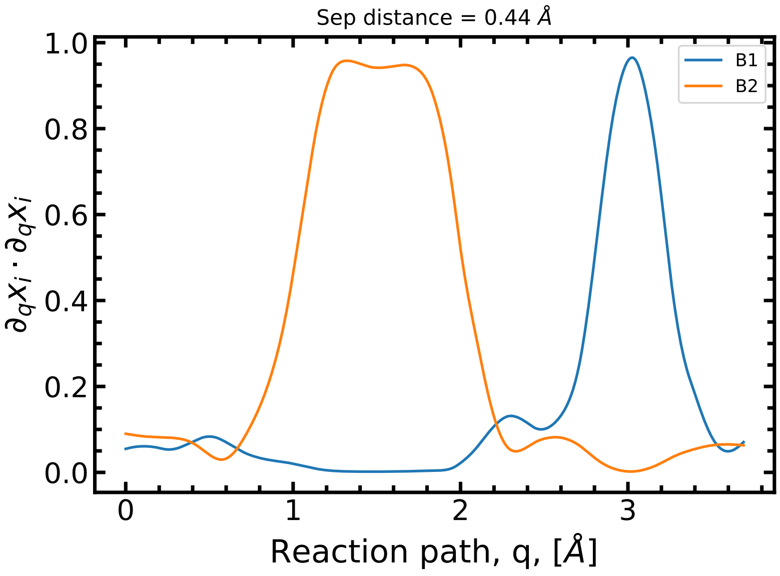

(a) Double proton transfer reaction path at 0.44 Å

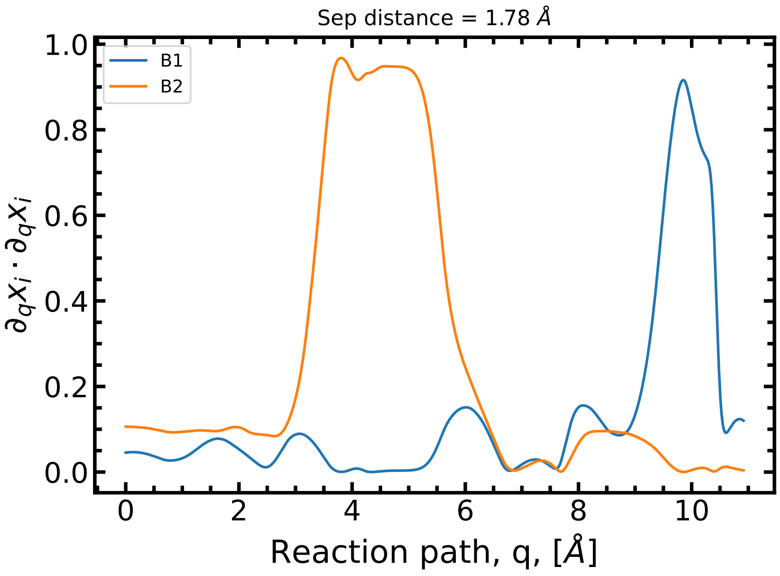

(b) Double proton transfer reaction path at 1.78 Å

Supplementary Figure 7: Evaluating the dot product of the derivative of the Cartesian vector with respect to the overall reaction coordinate at a fixed separation distance of 0.44 Å and 1.78 Å.

SUPPLEMENTARY NOTE 4: FURTHER NOTES ON PROTON TRANSFER ASYNCHRONICITY

We determine the asynchronicity to analyse further how the proton transfer mechanism changes during the base dissociation process. As a concept, asynchronicity is defined by a slight separation of the double proton transfer, i.e. one proton transfers, other heavy ions rearrange and then the second proton transfers.

To evaluate the derivatives shown in the methods section "Proton Transfer Asynchronicity", we first pass the Cartesian coordinate vectors into a Savitzky–Golay filter to suppress any spurious noise introduced by the uncertainty in the path which is inherent to the machine-learning approach of finding the reaction path. The filtered Cartesian coordinate vector and the reaction path are then interpolated using cubic splines.

In the top panel of Fig. 7, the first peak demonstrates B2 transferring before the second peak corresponding to

B1. The distance between each peak is used to determine the asynchronicity. In the second panel, the two prominent peaks are observed, along with some additional oscillations of the atoms rearranging. However, here the reaction path is much longer, and there is a clear separation between the two transfer peaks. The separation between the peaks demonstrates the large asynchronicity of the transfer process.
