# Supplementary material for: Proton transfer during DNA strand separation as a source of mutagenic guanine-cytosine tautomers
Source: Commun Chem. 2022 Nov 5;5:144. doi: 10.1038/s42004-022-00760-x (PMC9814255; doi:10.1038/s42004-022-00760-x)
Supplement: Supplementary file 1 — Winokan_PR File [file 42004_2022_760_MOESM1_ESM.pdf]

nature portfolio

Peer Review File

Proton Transfer During DNA Strand Separation as a Source of Mutagenic Guanine-Cytosine Tautomers

Open Access This file is licensed under a Creative Commons Attribution 4.0 International License, which permits use, sharing, adaptation, distribution and reproduction in any medium or format, as long as you give appropriate credit to the original author(s) and the source, provide a link to the Creative Commons license, and indicate if changes were made. In the cases where the authors are anonymous, such as is the case for the reports of anonymous peer reviewers, author attribution should be to 'Anonymous Referee' followed by a clear attribution to the source work. The images or other third party material in this file are included in the article's Creative Commons license, unless indicated otherwise in a credit line to the material. If material is not included in the article's Creative Commons license and your intended use is not permitted by statutory regulation or exceeds the permitted use, you will need to obtain permission directly from the copyright holder. To view a copy of this license, visit http://creativecommons.org/licenses/by/4.0/.

Reviewers' comments:

Reviewer #1 (Remarks to the Author):

The manuscript entitled "Proton Transfer During DNA Strand Separation as a Source of Mutagenic Guanine-Cytosine Tautomers" provides very interesting information about how the distance between DNA strands influence, in terms of energetics and feasibility, the proton transfer mechanism in a guanine-cytosine base pair. The presented results are relevant and of broad interest for the DNA community. Beyond, the manuscript is well written and is easy to follow. Therefore, I can recommend the paper for publication in Communications in Chemistry with some minor comments.

Minor issues:

1. If I correctly understood, at least for the DFT part, the authors just consider a guanine-cytosine base pair; however, I wonder how stacking should affect this proton transfer mechanism specially for those cases in which an important internal rotation takes place.

2. It is surprising that the preferred PT mechanisms involved B1. From the literature and the authors work this seems to be well assessed for the PT mechanism in the ground state. Interestingly it differs from the excited state mechanism in "proton coupled electron transfer" processes in which the preferred involved proton is B2. Can the authors comment a bit on that?

Reviewer #2 (Remarks to the Author):

In this manuscript authors tried to solve a very important biological problem. Will Guanine-Cytosine tautomers lead to the point mutation during DNA replication process? Authors presented the double proton transfer pathway during the unzipping of DNA duplex by using Density Functional Theory and also performed Molecular Dynamics to simulate opening angles.
However, I felt that this manuscript failed to provide the readers the clear answer for the question which authors raised. So I recommend not to publish the manuscript and Authors might rewrite the manuscript to focus on the double proton transfer mechanism which is the strong point of this manuscript.

Reviewer #3 (Remarks to the Author):

The authors analyze the effect of DNA strand separation on the possible formation and survival of Guanine-Cytosine rare tautomer (G*C*) using DFT calculations and classical molecular dynamics simulations. In the DFT calculations they include (only) the two bases, G and C, and use an implicit continuum solvation model with a dielectric factor of 8 to describe the effect of the DNA environment (rest of DNA, water molecules and counter-

ions). In the MD simulations they use the CHARMM36 force-field and a B-DNA model with 14 base pairs, with explicit solvent.

The authors find that the reverse (and forward) barriers for proton transfer increase drastically as a function of the separation distance (B2 proton transfer in Fig. 5c). This is the expected result in terms of chemical bonding. A larger reverse barrier implies a longer lifetime for the rare tautomer, a result that might increase the probability of spontaneous mutations in DNA.

The authors also estimate the base pair separation speed by means of steered MD simulations; they find a separation speed two orders of magnitude faster than previously considered. Thus, the separation of a base pair takes place on the order of pico-seconds.

The authors conclude that these results show that the process of DNA strand separation should be considered in more detail to determine the possible appearance of G*C* tautomers responsible for DNA spontaneous mutations.

I think these findings are interesting. My main comment is the following:

The reverse barrier (G*C* -> GC) increases rapidly as function of the separation distance between G and C; this yields a drastic increase in the lifetime of the mutagenic tautomer (G*C*). On the other hand, the forward barrier (GC -> G*C*) also increases as a function of separation distance. Notice also that recent QM/MM simulations show that the G*C* is unstable in the DNA biomolecular environment, see ref. [6]. Thus, although the (survival) lifetime of G*C* increases dramatically during the process of DNA strand separation, the probability of “trapping” a G*C* tautomer is probably extremely low: at the equilibrium distance there is no metastable G*C* state, while for larger separation distances there is a very large energy barrier.

I recommend the authors to present a discussion of this point in the manuscript.

Response to reviewer reports: Proton Transfer During DNA Strand Separation as a Source of Mutagenic Guanine-Cytosine Tautomers

[redacted]

September 6, 2022

Thank you for giving us the opportunity to submit a revised draft of the manuscript "Proton Transfer During DNA Strand Separation as a Source of Mutagenic Guanine-Cytosine Tautomers". We thank all the referees for their comprehensive reports. The level of detail in the referee's responses is appreciated, as is their time and effort to provide insightful comments and suggestions. In light of the concerns raised by the referees, we have made significant improvements to the content and structure of the paper and have incorporated all of their suggestions. They raised the requirement of a more detailed discussion of the preferred double proton transfer mechanism, the influence of stacking, and the probability of 'trapping' the G*C* tautomer. These issues have now been carefully addressed. We have typeset parts of the referees' comments to clarify our responses. We have highlighted the changes we have made to address their comments.

Outlined below are our responses and changes to the paper in line with their suggestions. Our response is formatted in the following way:

> *The referees' comments are in italics and coloured blue.*

Response Our response is written unindented adjacent the bold lettering.

- Explanation and location of the changes are shown here.
  The changes made to the revised manuscript are in red.

1 Referee 1

> *The manuscript entitled "Proton Transfer During DNA Strand Separation as a Source of Mutagenic Guanine-Cytosine Tautomers" provides very interesting information about how the distance between DNA strands influence, in terms of energetics and feasibility, the proton transfer mechanism in a guanine-cytosine base pair. The presented results are relevant and of broad interest for the DNA community. Beyond, the manuscript is well written and is easy to follow. Therefore, I can recommend the paper for publication in Communications in Chemistry with some minor comments.*

Response We thank Referee 1 for their positive interest in the manuscript and their insightful review.

1.1 Point 1

> *If I correctly understood, at least for the DFT part, the authors just consider a guanine-cytosine base pair; however, I wonder how stacking should affect this proton transfer mechanism specially for those cases in which an important internal rotation takes place.*

Response We thank the referee for highlighting this. We agree that explicitly including more degrees of freedom into our model could be of great interest. This point is also raised by Referee 3, and some excellent work has been conducted on this topic by Ref. 6 and 9. However, exploring the role of the stacking interactions in enabling or (disabling) double proton transfer would increase the complexity of the model and the computational cost prohibitively. In particular, this is attributed to the transition state calculations which strongly and non-linearly depend on the degrees of freedom of the system. Therefore, we have had the resources and capability only to conduct one additional transition state search calculation, see the changes to the manuscript itemised below. The calculation was performed at ~ 0.39 Å separation distance, thus its PES is distinct from those reported in Ref. 6 and 9, which were conducted at the equilibrium distance. The initial conditions for our additional calculation are deduced from the MM-MD run presented in the section entitled "MD opening angles". In the calculation, we include only the rung below in addition to the separating base. Since the base above is assumed to be fully separated by the action of the helicase enzyme, we believe this approximation to be justified. Furthermore, we assume a separation of timescales, whereby the base which begins to separate relaxes, while the base below remains unchanged by the base separation. This allows a compromise between computational expense by 'freezing' the bottom bases but directly includes the stacking effect. However, we point out that further work is required to determine how the arrangement of the other bases and the rungs changes when helicase is introduced. This is out of the scope of this work, and we are currently working on addressing this in a separate study. Therefore, following Referee 1's comment, we have made the following changes.

- We have added an additional transition state search calculation which includes the stacking interaction of the base below. See pages 6 and 7.
  Including a more comprehensive description of the cellular environment (stacking and solvent effects) has recently been suggested to alter the reaction asymmetry [1] such that the tautomeric state vanishes on the free energy surface. Such interactions could avert the tautomer's formation and prevent it from potentially leading to a mutation. Gheorghiu *et al.* [2] suggest coupling to the larger environment offers a diverse ensemble of reaction pathways. To investigate this, we took a snapshot from an MD run presented above and re-determined the reaction path when including the rung below the separating base (pair G2-C2 in Fig 1). In the calculation we assume that the base above is fully separated and thus, we can omit it from our simulation system as it no longer introduces stacking interactions with the base in question.

Furthermore, we assume a separation of timescales whereby the base which begins to separate would relax, while the base below would remain largely unchanged by the base separation. This assumption is further justified in Supplementary Note 4. Using the same methods as described before, at 0.39 Å separation distance the first energy barrier for proton transfer has an energy of 1.08 eV. This can be compared to 1.06 eV at the same distance but with two isolated bases. The second barrier has an energy of 0.098 eV (vs 0.120 eV with the isolated base pair), with a reaction asymmetry of 0.467 eV (vs 0.462 eV with the isolated base pair). The second reaction barrier shows the largest difference to the PES of the single base pair proton transfer, with a change of 0.02 eV. This finding is consistent with Das *et al.* [3] who conducted MD simulations and showed that adjacent base pair stacking modifies the proton transfer profile on the order of 0.04 eV.

- We added further discussion on stacking interactions and their impact on the proton transfer mechanism. See page 7.
  While stacking interactions show only a minor contribution to the proton transfer energy path, in this approach, we do not explore other thermodynamically valid conformations (akin to Gheorghiu *et al.* [2]) that may depend more on stacking interactions. Furthermore, it is unclear if this would translate to a system in contact with helicase and warrants further investigation.

- We clarified the language used in the "MD opening angles" section to highlight that the rotation is in agreement with the MD result, which includes stacking interactions. However, it does not provide information on how the proton transfer changes due to stacking. See page 4.
  Fig. 2) which predicts B3 to stretch up to ~ 1.7 Å (relative to B1) before B1 is dilated. This suggests that the bond angles of the DFT calculations on the single base pairs are consistent with the MD picture, which includes the larger structure. However, it is unclear how the larger structure influences the proton transfer.

- We have produced an additional figure from our MD trajectories revealing that base pair 2 remains in equilibrium separation while base pair 1 undergoes a separation event. This justifies our constraints on the second base pair in the DFT calculations of the stacking interaction. This figure and an additional explanation are included in Supplementary Note 4 (page 7).
  During our quantum mechanical calculations where the effect of stacking interactions between DNA base pairs on the double proton transfer was investigated, the base pair below the base pair of interest was assumed to be frozen during the separation event in the base pair above it. The resulting reaction profile is shown in Fig. 6. This assumption was not taken lightly, and backed up by our molecular dynamics simulations, in which we can see a clear separation of timescales between the opening of one base pair, and the next. This can be deduced from Fig. 7 where the separation of base pair 2 (plotted on the y-axis) remains near-zero during separation events in base pair 1 (x-axis).

1.2 Point 2

It is surprising that the preferred PT mechanisms involved B1. From the literature and the authors work this seems to be well assessed for the PT mechanism in the ground state. Interestingly it differs from the excited state mechanism in "proton coupled electron transfer" processes in which the preferred involved proton is B2. Can the authors comment a bit on that?

Response Thanks for the suggestion. We agree that a comparison to proton-coupled electron transfer is pertinent and would add to our findings.

- In light of your point, we have added further discussion on comparing our simulations on the ground state to proton-coupled electron transfer. See the new discussion on page 7.
  On the other hand, proton-coupled electron and hole transfer (PCET) is a prominent feature of radiation-induced excited state dynamics and subsequent DNA damage [4]. The current theoretical framework of PCET has successfully treated these types of problems [5,6]. For example, applying PCET to the excited state dynamics of the one-electron oxidation of G-C decouples the proton and electron transfer from the middle hydrogen bond N of G (B2 in our labelling) to the N on C [4]. Similarly, a PCET excited-state deactivation mechanism for G-C has been proposed from experiment and theory (see review Kumar *et al.* [4]). Furthermore, femtosecond transient absorption spectroscopy suggests that the excited state PCET between the DNA stands has a pronounced deuterium isotope effect [7]. These findings match the transition state calculations presented in this paper, as we determine the same B2 proton transfer pathway.

2 Referee 2

> *In this manuscript authors tried to solve a very important biological problem. Will Guanine-Cytosine tautomers lead to the point mutation during DNA replication process? Authors presented the double proton transfer pathway during the unzipping of DNA duplex by using Density Functional Theory and also performed Molecular Dynamics to simulate opening angles. However, I felt that this manuscript failed to provide the readers the clear answer for the question which authors raised. So I recommend not to publish the manuscript and Authors might rewrite the manuscript to focus on the double proton transfer mechanism which is the strong point of this manuscript.*

Response We thank Referee 2 for their honest review. We have decided to split the reviewers comments into two points which we will now individually address.

2.1 Point 1

> *. . . However, I felt that this manuscript failed to provide the readers the clear answer for the question which authors raised . . .*

Response We thank you for your comment and we agree that there is still a uncertainty in the understanding of how mutations arise, but it is clear that the helicase and the DNA strand separation dynamics are a critical part of the process. We believe that we have made changes to our manuscript which reflect this understanding, in particular that our work addresses only a small, but important, factor in the field of genetic mutations.

- We have added more discussion on contrasting literature to exemplify the issue of the conflict within the literature. See also the discussion and changes regarding point 1 from reviewer 1. See pages 6 and 7.
  Including a more comprehensive description of the cellular environment (stacking and solvent effects) has recently been suggested to alter the reaction asymmetry [1] such that the tautomeric state vanishes on the free energy surface. Such interactions could avert the tautomer's formation and prevent it from potentially leading to a mutation. Gheorghiu *et al.* [2] suggest coupling to the larger environment offers a diverse ensemble of reaction pathways.

- We have rewritten the end of the abstract. See page 1.
  ...In addition, molecular dynamics simulations indicate that the relevant stand separation time is two orders of magnitude quicker than previously thought. Our results demonstrate that the unwinding of DNA by the helicase could simultaneously slow the formation but significantly enhance the stability of tautomeric base pairs and provide a feasible pathway for spontaneous DNA mutations.

- We have rewritten the conclusion to answer this point directly. See page 8.
  Consequently, we determine that a direct comparison between the biological timescale and the lifetime of the tautomeric state is misleading when making assertions about tautomeric populations becoming mutations. Furthermore, we suggest that the method of determining the proton transfer rate kinetics needs to be revised since the system is out of equilibrium. Instead, a well-parameterised time-dependent kinetic model is required to describe the low initial population and its subsequent trapping.

  In summary, the work here only scratches the surface of describing the biology involved in producing the mutations from the proton transfer mechanism. However, we have gone further than the status quo and laid the path forward to accurately determining the mutation mechanism. Finally, to fully answer whether G-C tautomers lead to the point mutation during DNA replication, we underscore the requirement to combine a time-dependent kinetic model to resolve the competing biological splitting timescale with calculations of the interaction between helicase and the tautomer.

2.2 Point 2

> *...rewrite the manuscript to focus on the double proton transfer mechanism which is the strong point of this manuscript.*

Response Thanks for the comment. We believe that our paper attempted to address the issue of how the (double) proton transfer changes when the induced strand separation is introduced. However, as you highlight, it would be prudent to clarify this further.

- We want to raise the discussion of the proton transfer mechanism found on pages 6 and 7. Here, we discuss that this is counter to some previous papers on this work but also agrees with some more recent work. While we raised the double proton transfer mechanism, we need to expand the analysis and discussion further. To further clarify the comparison, we have added more discussion on this topic. We added further comparison to the GS method with excited state proton-coupled electron approaches. See page 7.
  On the other hand, proton-coupled electron and hole transfer (PCET) is a prominent feature of radiation-induced excited state dynamics and subsequent DNA damage [4]. The current theoretical framework of PCET has successfully treated these types of problems [5, 6]. For example, applying PCET to the excited state dynamics of the one-electron oxidation of G-C decouples the proton and electron transfer from the middle hydrogen bond N of G (B2 in our labelling) to the N on C [4]. Similarly, a PCET excited-state deactivation mechanism for G-C has been proposed from experiment and theory (see review Kumar *et al.* [4]). Furthermore, Femtosecond transient absorption spectroscopy suggests that the excited state PCET between the DNA stands has a pronounced deuterium isotope effect [7]. These findings match the transition state calculations presented in this paper, as we determine the same B2 proton transfer pathway.

- We have added further analysis on the asynchronicity of the transfer in the main body of the manuscript. We believe this furthers our analysis of how the double proton transfer changes when the induced strand separation is introduced. See the new Fig. 6 (top of page 7) and text (pages 6 and 7).
  Fig. 6 displays the asynchronicity as a function of the separation distance induced between the DNA bases. Asynchronicity is a measure of the separation between the two proton transfer events; further detail can be found in the methods section and Supplementary Note 3. Here we use asynchronicity to quantify how the proton transfer mechanism changes during the base dissociation process. Fig. 6 demonstrates that the double proton transfer initially has some concurrence indicated by a low asynchronicity value. However, as the bases are further apart, the first and second proton transfer becomes an increasingly separate event. The disconnection of the two proton transfer events along the reaction path could lead to a distribution of outcomes of product states. Furthermore, the increased asynchronicity indicates an increased localisation of the single proton transfer. Consequently, the single proton transfer could also occur along with the double proton pathway. However, due to the prohibitively significant initial forward reaction barrier, the population of either product becomes increasingly unlikely as the DNA base is further separated by the replication machinery.

- We have added a description of the methods for analysing the asynchronicity. See the "Proton Transfer Asynchronicity" subsection in the methods on page 9.
  To further analyse how the proton transfer mechanism changes during the base dissociation process we determine the asynchronicity (α) of the double proton transfer. As a concept, asynchronicity is defined by a slight separation of the two proton transfers, i.e. one proton transfers, other heavy ions rearrange and then the second proton transfers. We formally define asynchronicity as

$$\alpha = |\alpha_{\mathrm{B1}} - \alpha_{\mathrm{B2}}| \,. \tag{1}$$

  Where,

$$\alpha_i = \mathrm{argmax}\left(\frac{\partial x_i}{\partial q_{\mathrm{IRC}}} \cdot \frac{\partial x_i}{\partial q_{\mathrm{IRC}}}\right). \tag{2}$$

  Here x_i is the Cartesian vector of atom i and q_{IRC} is the reaction coordinate. The partial derivative of the Cartesian vector tracks the motion of, say, B1 or B2 along the reaction coordinate. The dot product normalises the B1 or B2 motion relative to the collective rearrangement of all atoms. If there is no motion of atom i, it does not contribute to the reaction path, then $\alpha_i \to 0$.If $\alpha_{\mathrm{B1}} \sim \alpha_{\mathrm{B2}}$ the protons transfer at the same point on the reaction coordinate, thus the process is synchronous. While when $\alpha > 0$, one proton moves before another.

- We have added further analysis on the asynchronicity of the transfer in the supporting information document on pages 6 onwards.
  See "Supplementary Note 3: Further Notes on Proton Transfer Asynchronicity". Here, two new figures have been added and further supporting methods and analysis.

3 Referee 3

*The authors analyze the effect of DNA strand separation on the possible formation and survival of Guanine-Cytosine rare tautomer (G*C*) using DFT calculations and classical molecular dynamics simulations. In the DFT calculations they include (only) the*

two bases, G and C, and use an implicit continuum solvation model with a dielectric factor of 8 to describe the effect of the DNA environment (rest of DNA, water molecules and counter-ions). In the MD simulations they use the CHARMM36 force-field and a B-DNA model with 14 base pairs, with explicit solvent.

The authors find that the reverse (and forward) barriers for proton transfer increase drastically as a function of the separation distance (B2 proton transfer in Fig. 5c). This is the expected result in terms of chemical bonding. A larger reverse barrier implies a longer lifetime for the rare tautomer, a result that might increase the probability of spontaneous mutations in DNA.

The authors also estimate the base pair separation speed by means of steered MD simulations; they find a separation speed two orders of magnitude faster than previously considered. Thus, the separation of a base pair takes place on the order of pico-seconds.

*The authors conclude that these results show that the process of DNA strand separation should be considered in more detail to determine the possible appearance of G*C* tautomers responsible for DNA spontaneous mutations.*

I think these findings are interesting.

Response We thank Referee 3 for their interest in the manuscript.

3.1 Point 1

*The reverse barrier (G*C* → GC) increases rapidly as function of the separation distance between G and C; this yields a drastic increase in the lifetime of the mutagenic tautomer (G*C*). On the other hand, the forward barrier (GC → G*C*) also increases as a function of separation distance. Notice also that recent QM/MM simulations show that the G*C* is unstable in the DNA biomolecular environment, see ref. [6]. Thus, although the (survival) lifetime of G*C* increases dramatically during the process of DNA strand separation, the probability of "trapping" a G*C* tautomer is probably extremely low: at the equilibrium distance there is no metastable G*C* state, while for larger separation distances there is a very large energy barrier.*

I recommend the authors to present a discussion of this point in the manuscript.

Response Thanks for highlighting this; we agree, this is an excellent point to add to the discussion.

- We have added the following new sentences to the conclusion on page 8.
  ...In particular, we observe a linear dependence of the energy of the first barrier on the separation distance. Consequently, the G-C tautomeric state becomes more stable as the hydrogen bonds break. The overall reverse barrier of the proton transfer (G*C* → GC) increases rapidly as a function of the separation distance between G and C; this yields a drastic increase in the lifetime of the potentially mutagenic tautomer (G*C*). On the other hand, the forward barrier (GC → G*C*) also increases as a function of separation distance. Thus, although the survival lifetime of G*C* increases dramatically during the process of DNA strand separation, the overall probability of "trapping" a G*C* tautomer is probably extremely low. At the equilibrium distance, there is debate over a metastable G*C* state [2], while there is a substantial energy barrier for larger separation distances. ...

- We have modified the last section of the abstract on page 1.
  ...In addition, molecular dynamics simulations indicate that the relevant strand separation time is two orders of magnitude quicker than previously thought. Our results demonstrate that the unwinding of DNA by the helicase could simultaneously slow the formation but significantly enhance the stability of tautomeric base pairs ...mutations.

References

[1] Diego Soler-Polo, Jesús I Mendieta-Moreno, Daniel G Trabada, Jesús Mendieta, and José Ortega. Proton transfer in guanine-cytosine base pairs in b-dna. *Journal of chemical theory and computation*, 15(12):6984–6991, 2019.

[2] A Gheorghiu, PV Coveney, and AA Arabi. The influence of base pair tautomerism on single point mutations in aqueous dna. *Interface focus*, 10(6):20190120, 2020.

[3] Susanta Das, Kwangho Nam, and Dan Thomas Major. Rapid convergence of energy and free energy profiles with quantum mechanical size in quantum mechanical–molecular mechanical simulations of proton transfer in dna. *J. Chem. Theory Comput.*, 14(3):1695–1705, 2018.

[4] Anil Kumar and Michael D Sevilla. Proton-coupled electron transfer in dna on formation of radiation-produced ion radicals. *Chemical reviews*, 110(12):7002–7023, 2010.

[5] David R Weinberg, Christopher J Gagliardi, Jonathan F Hull, Christine Fecenko Murphy, Caleb A Kent, Brittany C Westlake, Amit Paul, Daniel H Ess, Dewey Granville McCafferty, and Thomas J Meyer. Proton-coupled electron transfer. *Chemical Reviews*, 112(7):4016–4093, 2012.

[6] Robin Tyburski, Tianfei Liu, Starla D Glover, and Leif Hammarström. Proton-coupled electron transfer guidelines, fair and square. *Journal of the American Chemical Society*, 143(2):560–576, 2021.

[7] Kimberly de La Harpe, Carlos E Crespo-Hernández, and Bern Kohler. Deuterium isotope effect on excited-state dynamics in an alternating gc oligonucleotide. *Journal of the American Chemical Society*, 131(48):17557–17559, 2009.

REVIEWERS' COMMENTS:

Reviewer #1 (Remarks to the Author):

The authors have addressed all the minor comments suggested initially, even performing additional calculations to provide insights about the stacking effect into the manuscript conclusions. Furthermore, the section concerning comparison with PCET has been expanded. I can now recommend the paper for publication.

Reviewer #2 (Remarks to the Author):

With the addition of base stacking interactions, authors undoubtedly strengthen their double proton transfer mechanism during the strand separation. Also they revised the conclusion to clarify the limitation of their study for this important and complicated biological problem. Now I believe that this paper provided new insight to the field and will attract more interest to understand the problem further. I recommend this paper for publication.

Reviewer #3 (Remarks to the Author):

The authors have considered the main comment in my previous Referee report and have added a discussion in the manuscript.
I think that the results presented in the manuscript are interesting and I recommend publication in CommsChem.

Regarding the new Figure 6 (asynchronicity), I am not sure if the vertical axis is correct.
